# QTAIM analysis dataset for non-covalent interactions in furan clusters

**DOI:** 10.1016/j.dib.2021.107766

**Published:** 2021-12-24

**Authors:** Alhadji Malloum, Jeanet Conradie

**Affiliations:** a Department of Chemistry, University of the Free State, PO BOX 339, Bloemfontein 9300, South Africa; b Department of Physics, Faculty of Science, University of Maroua, PO BOX 46, Maroua, Cameroon; c Department of Chemistry, UiT - The Arctic University of Norway, N-9037 Tromsø, Norway

**Keywords:** Furan clusters, Non-covalent interactions, QTAIM analysis, Heterocyclic molecules, Benzene clusters

## Abstract

Furan clusters are very important to understand the dynamics and properties of the furan solvent. They can be used combined with quantum cluster equilibrium theory to theoretically determine the thermodynamics properties of the furan solvent. To understand the structures of the furan clusters, one needs to understand the non-covalent interactions that hold the furan molecules together. In this paper, we have provided the data necessary to understand the non-covalent interactions in furan clusters. Firstly, the structures of the furan clusters have been generated using classical molecular dynamics as implemented in the ABCluster code. Secondly, the generated structures have been fully optimized at the MP2/aug-cc-pVDZ level of theory. The optimized Cartesian coordinates of all the investigated structures are reported in this work to enable further investigations of the furan clusters. These Cartesian coordinates will save computational time for all further investigations involving the furan clusters. Thirdly, to understand the nature of the non-covalent interactions in furan clusters, we have performed a quantum theory of atoms in molecule (QTAIM) analysis using AIMAll program. Using QTAIM, we have provided the critical points, bond paths and their related properties for all the investigated structures. These data can be used to identify and classify the non-covalent interactions in furan clusters. The reader can refer to the original article for further information and discussion of the data provided herein Malloum and Conradie (2022) [1].

## Specifications Table

**Table:** 

Subject	Chemistry
Specific subject area	Physical and Theoretical Chemistry
Type of data	Figure
	Table
How data were acquired	Geometrical structures data have been obtained using the Gaussian 16 quantum Chemistry program. The data for the analysis using quantum theory of atoms in molecules have been generated by AIMAll code.
Data format	Analyzed
	Raw
Parameters for data collection	Raw data (optimized Cartesian coordinates of the structures) are extracted directly from the Gaussian output files. Analyzed data (description of critical points and their related figures) were obtained from the AIMAll program.
Description of data collection	Optimization of the geometries have been performed using the resources of the Center of High Performance Computing (CHPC), South Africa. The QTAIM analysis has been performed in our laboratory (Physical Chemistry Laboratory of the Department of Chemistry, University of the Free State).
Data source location	Institution: Department of Chemistry, University of the Free State
	City/Town/Region: Bloemfontein
	Country: South Africa
Data accessibility	With the article
Related research article	Alhadji Malloum and Jeanet Conradie, Structures, Binding Energies and Non-Covalent Interactions of Furan Clusters, J. Mol. Graph. Model. 111 (2022) 108102 [1]. https://doi.org/10.1016/j.jmgm.2021.108102

## Value of the Data

• The critical points and bond paths of the structures of the furan tetramer give visual understanding of the non-covalent interactions in furan clusters.
• Cartesian coordinates data of the structures of the furan clusters from dimer to tetramer will be useful for further investigations on furan clusters. For instance, this data can be used directly to calculate the properties of furan clusters without prior optimizations.
• Data in tables reporting the properties of bond critical points can be used to quantify the strength and determine the nature of non-covalent interactions in furan clusters.
• The data reported in this work can be used combined with quantum cluster equilibrium theory to determine the thermodynamics properties of furan solvent.
• The optimized Cartesian coordinates can be used for further investigations involving proton transfer, ions transfer, proton and ions solvations taking place in furan solvent.

## 1 Data Description

**Fig. 1:**
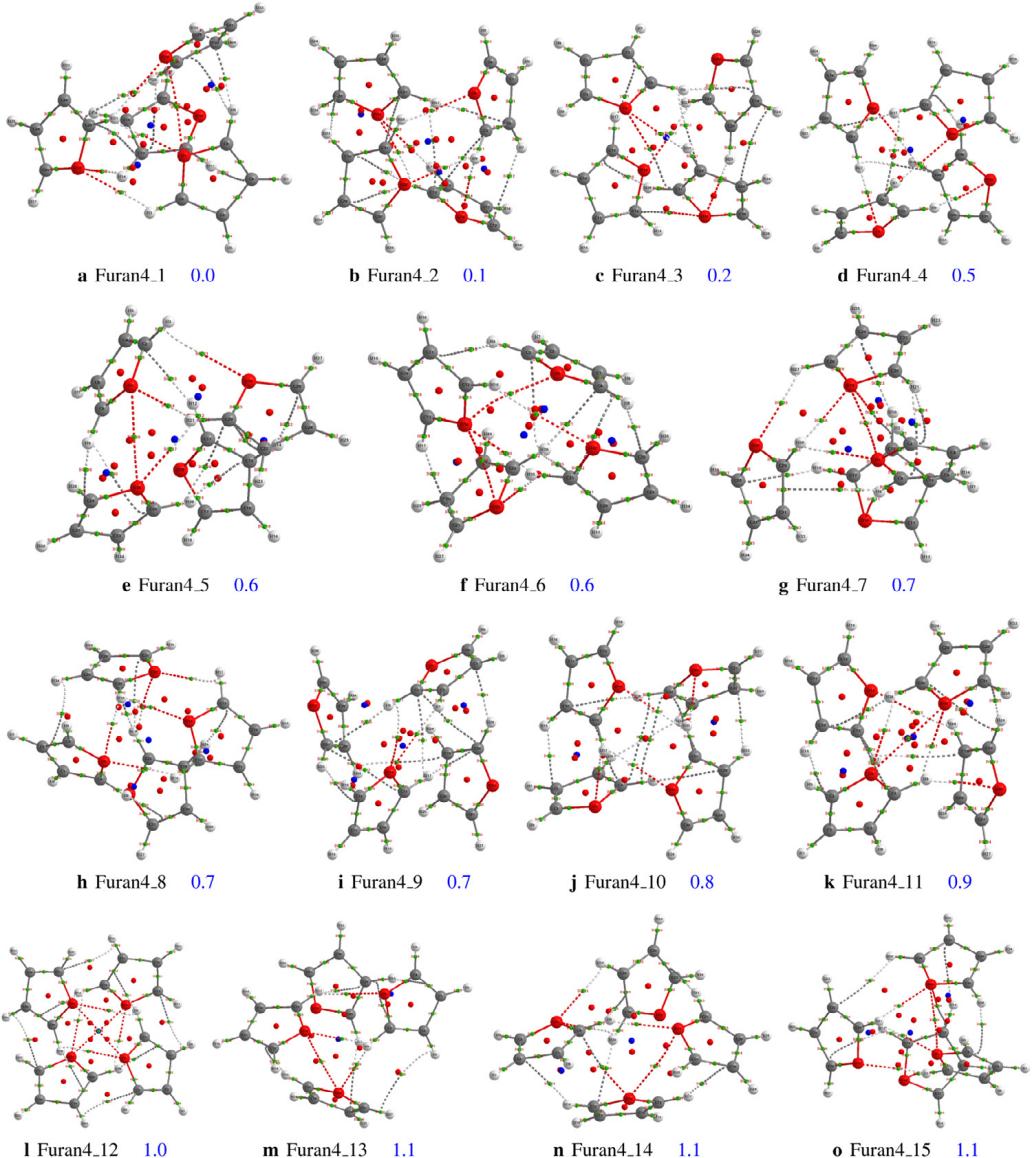
Critical points and bond paths of the structure of the furan tetramer within 1.0 kcal/mol. Bond critical points (BCP) are reported in green color, Ring critical points (RCP) are in red color while cage critical points (CCPs) are in blue color. Isomers **Furan4_1** to **Furan4_4** are from the original research paper.

We have reported in Fig. 1 the structures of the furan tetramer and their relative energy as optimized at the MP2/aug-cc-pVDZ level of theory. In addition, Bond critical points (BCP), Ring critical points (RCP) and cage critical points (CCPs) are reported therein. In Fig. 1, covalent bonds are represented by solid lines while non-covalent interactions are represented by dash lines. Fig. 1 can be used to determine the possible non-covalent interactions of the furan clusters. Furthermore, we have reported the properties of the bond critical points of the corresponding structures of the furan dimer to tetramer in the supplementary file (excel tables). The structures of the furan dimer are reported in the original research paper, and the coordinates of the dimers are provided in the supporting information. The BCP properties reported in these tables are described as follows: the name of the bond critical point (**name**), the two atoms involved in the bond critical point (**Atoms**), the electron density at the bond critical point (ρ), the Laplacian of the electron density at the bond critical point ($\nabla^{2}\rho$), the ellipticity of the bond (**Ellipticity**), the electron kinetic energy density at the bond critical point (**K**) and the difference between the bond path length with a corresponding geometric bond length (**BPL-GBL_I**). Using the data reported in these tables one can quantify the strength of the non-covalent interactions. In addition, these tables can also be used to classify the different types of non-covalent interactions of the furan clusters. Furthermore, the Cartesian coordinates of all the investigated structures of the furan clusters from dimer to tetramer are provided in the supplementary material. These Cartesian coordinates have been initially generated using ABCluster code followed by full optimizations at the MP2/aug-cc-pVDZ level of theory (see further description in the methodological section).

## 2 Experimental Design, Materials and Methods

Initially, the geometries of the furan clusters have been generated using classical molecular dynamics simulations as implemented in ABCluster code. ABCluster performs a global optimization to identify local and global minima energy structures on the potential energy surfaces (PESs) of molecular clusters. ABCluster uses a potential that consider electrostatics and Lenard–Jones interactions. The potential used by ABCluster is given by:

(1) $$U=\sum_{I=1}^{N}\sum_{J<I}^{N}\sum_{i_{I}}\sum_{j_{J}}\left(\frac{e^{2}}{4\pi \epsilon_{0}}\frac{q_{i_{I}}q_{j_{J}}}{r_{i_{I}j_{J}}}+4\epsilon_{i_{I}j_{J}}\left({\left(\frac{\sigma_{i_{I}j_{J}}}{r_{i_{I}j_{J}}}\right)}^{12}-{\left(\frac{\sigma_{i_{I}j_{J}}}{r_{i_{I}j_{J}}}\right)}^{6}\right)\right).$$

where I and J are the indices of the molecules, $i_{I}$ and $j_{J}$ are the indices of the atoms in molecules I and J, respectively. $r_{i_{I}j_{J}}$ is the distance between atom $i_{I}$ and $j_{J}$. There are three parameters that needs to be set in ABCluster: the scout limit $g_{\text{limit}}$, the size of the population of trial solutions SN and the maximum cycle number $g_{\max}$. In the present investigations, we used $g_{\text{limit}}=4$, SN=60 and $g_{\max}=10,000$. ABCluster performs the global optimization using the bee colony algorithm. It tries to mimic the foraging behavior of bees in nature to locate the best nectar. The ABCluster code has been used successfully in our previous work to generate initial structures for further optimization [2], [3], [4], [5], [6]. The reader can refer to those works or the original works of Zhang and Dolg [7], [8] for further details on the generation of the structures using ABCluster. The geometries generated using ABCluster have been fully optimized at the MP2/aug-cc-pVDZ level of theory. Frequencies calculation has been performed at the same level of theory to ensure true location of local/global minima. Optimization and frequencies calculations have been performed using Gaussian 16 suite of program. To ensure the accuracy of the optimization we used the **tight** option of the optimization.

We used the AIMAll [9] program to generated the data used for the quantum theory of atoms in molecule (QTAIM) analysis. It should be noted that AIMAll uses the formatted checkpoint file from the optimized structures at the MP2/aug-cc-pVDZ using Gaussian 16. QTAIM analyses the topology of the electron density of a given molecule to determine the critical points of the system. The roots of the first order derivatives of the electron density represent the critical points of the molecule. There are four critical points which are mainly used in QTAIM analysis: atom critical point (3, −3) ; bond critical points (3, −1); ring critical points (3, 1); and cage critical points (3, 3). These critical points are identified depending on the sign of the second order derivatives of the electron density, $\nabla^{2}\rho$. Two atoms critical points (ACPs) can be linked by a bond path (BP) along which lies a bond critical point (BCP). The BCP is located where the electron density has its minimum value along the bond path. The electron density and the second order derivatives of the electron density at a bond critical point can be used to identify the nature of the corresponding bonding [10], [11]. The topology of the electron density and the properties of bond critical points provide a universal description of bonding [12]. A positive value of $\nabla^{2}\rho$ is indicative of non-covalent interaction while negative value of $\nabla^{2}\rho$ is indicative of covalent bonding. Depending on the interval in which the value of ρ falls, one can identify the nature of the corresponding bonding. The range proposed for ρ and $\nabla^{2}\rho$ at a bond critical point for a hydrogen bond to exist, is 0.002-0.035 $ea_{0}^{-3}$ for ρ and 0.024-0.139 $ea_{0}^{-5}$ for $\nabla^{2}\rho$ [13].

## CRediT authorship contribution statement

**Alhadji Malloum:** Conceptualization, Methodology, Validation, Formal analysis, Investigation, Data curation, Writing – original draft, Visualization. **Jeanet Conradie:** Resources, Visualization, Writing – review & editing, Supervision, Funding acquisition, Project administration.

## Declaration of Competing Interest

The authors declare that they have no known competing financial interests or personal relationships which have, or could be perceived to have, influenced the work reported in this article.

## Appendix A Supplementary materials

Supplementary Data S1 Supplementary Raw Research Data. This is open data under the CC BY license http://creativecommons.org/licenses/by/4.0/ Supplementary Data S1

Application 2 Supplementary Data S1
