# Supplementary material for: QTAIM analysis dataset for non-covalent interactions in furan clusters
Source: Data Brief. 2021 Dec 24;40:107766. doi: 10.1016/j.dib.2021.107766 (PMC8718740; doi:10.1016/j.dib.2021.107766)
Supplement: Application 2 [file mmc2.pdf]

Cartesian coordinates of : Furan2_1

Atomic number (AN) and Cartesian coordinates

AN	X	Y	Z
C	-1.140351	0.281677	-1.281053
C	-1.683943	1.202933	-0.323689
C	-2.146658	0.437124	0.727675
C	-1.309117	-0.976752	-0.738404
O	-1.933718	-0.894917	0.480716
H	-0.681161	0.508057	-2.240550
H	-1.727308	2.288204	-0.391207
H	-1.077662	-1.979300	-1.087074
H	-2.637354	0.672476	1.668291
C	1.140347	0.281689	1.281048
C	1.683947	1.202934	0.323676
C	2.146665	0.437113	-0.727677
C	1.309111	-0.976745	0.738413
O	1.933717	-0.894924	-0.480707
H	0.681158	0.508079	2.240543
H	1.727325	2.288205	0.391189
H	1.077648	-1.979288	1.087091
H	2.637354	0.672453	-1.668299

Cartesian coordinates of : Furan2_2

Atomic number (AN) and Cartesian coordinates

AN	X	Y	Z
C	-2.040315	1.010777	-0.315317
C	-1.272419	0.932430	0.896477
C	-1.107594	-0.412357	1.158500
C	-2.286988	-0.293011	-0.698519
O	-1.728402	-1.167951	0.196956
H	-2.372242	1.905053	-0.839444
H	-0.881577	1.753229	1.493717
H	-2.823843	-0.742441	-1.529776
H	-0.603493	-0.966020	1.944691
C	1.338383	-1.017405	-0.800679
C	2.107082	-0.924650	0.409984
C	2.262364	0.424002	0.663248
C	1.084226	0.280987	-1.192965
O	1.648340	1.167016	-0.311394
H	1.005140	-1.916970	-1.313682
H	2.498187	-1.738485	1.017687
H	0.545248	0.720341	-2.026756
H	2.764641	0.988130	1.444683

Cartesian coordinates of : Furan2_3

```
 ---------------------------------------------------
 Atomic number (AN) and Cartesian coordinates
  AN         X            Y            Z
 ---------------------------------------------------
  C     -1.994253      1.096874      0.120664
  C     -1.282940      0.760477     -1.082675
  C     -1.161104     -0.614438     -1.082948
  C     -2.246964     -0.097757      0.763577
  O     -1.751081     -1.148404      0.034927
  H     -2.282492      2.085280      0.473506
  H     -0.909467      1.434435     -1.850390
  H     -2.740536     -0.358262      1.695888
  H     -0.721061     -1.326922     -1.774267
  C      2.276770      0.857819     -0.387871
  C      2.375428     -0.564263     -0.563542
  C      1.528993     -1.125141      0.373392
  C      1.376534      1.054299      0.641134
  O      0.925808     -0.146771      1.118544
  H      2.792626      1.637324     -0.945296
  H      2.983301     -1.108312     -1.284095
  H      0.978624      1.937548      1.132670
  H      1.266403     -2.146916      0.633826
 ---------------------------------------------------

Cartesian coordinates of :  Furan2_4

 ---------------------------------------------------
 Atomic number (AN) and Cartesian coordinates
  AN         X            Y            Z
 ---------------------------------------------------
  O      1.756932      1.157372     -0.000081
  C      2.056036      0.397896     -1.102905
  H      1.864626      0.864265     -2.065333
  C      2.547131     -0.833921     -0.718865
  H      2.861563     -1.633328     -1.386670
  C      2.547068     -0.833823      0.719041
  H      2.861452     -1.633136      1.386982
  C      2.055941      0.398046      1.102872
  H      1.864430      0.864539      2.065219
  O     -2.110563     -1.236529     -0.000016
  C     -1.062397     -0.349155     -0.000055
  H     -0.069877     -0.791030     -0.000090
  C     -1.534474      0.948076     -0.000034
  H     -0.921946      1.846918     -0.000059
  C     -2.969119      0.853129      0.000020
  H     -3.693228      1.665834      0.000049
  C     -3.264507     -0.495151      0.000029
  H     -4.192047     -1.061402      0.000062
 ---------------------------------------------------

Cartesian coordinates of :  Furan2_5

 ---------------------------------------------------
```

```
 Atomic number (AN) and Cartesian coordinates
  AN         X            Y            Z
 ---------------------------------------------------
  O     -2.036911     -1.194907      0.132852
  C     -3.211264     -0.523006     -0.090380
  H     -4.092863     -1.142447     -0.232268
  C     -2.993857      0.840074     -0.085106
  H     -3.749694      1.608449     -0.236927
  C     -1.587856      1.017942      0.156240
  H     -1.035568      1.951867      0.233338
  C     -1.052869     -0.248530      0.280990
  H     -0.054081     -0.629336      0.474258
  O      2.000218     -0.358862     -1.167738
  C      1.856718      0.933809     -0.735441
  H      1.546616      1.652368     -1.489061
  C      2.151208      1.027239      0.610560
  H      2.119975      1.933725      1.211742
  C      2.493485     -0.301829      1.037180
  H      2.780972     -0.630454      2.033777
  C      2.383588     -1.104186     -0.082220
  H      2.543268     -2.163089     -0.266701
 ---------------------------------------------------

Cartesian coordinates of :  Furan2_6

 ---------------------------------------------------
 Atomic number (AN) and Cartesian coordinates
  AN         X            Y            Z
 ---------------------------------------------------
  O     -1.680510     -0.701496     -0.000018
  C     -2.998309     -1.085872     -0.000014
  H     -3.180473     -2.157193     -0.000027
  C     -3.820739      0.021368      0.000021
  H     -4.908968      0.009324      0.000040
  C     -2.950201      1.166105      0.000003
  H     -3.230986      2.217469      0.000007
  C     -1.661988      0.673839     -0.000001
  H     -0.676335      1.131623     -0.000003
  O      1.680510      0.701497     -0.000003
  C      1.661987     -0.673839      0.000006
  H      0.676335     -1.131622      0.000008
  C      2.950201     -1.166105      0.000017
  H      3.230985     -2.217470      0.000030
  C      3.820739     -0.021369     -0.000005
  H      4.908968     -0.009325     -0.000010
  C      2.998310      1.085871     -0.000004
  H      3.180475      2.157193     -0.000010
 ---------------------------------------------------


Cartesian coordinates of :  Furan3_1

 ---------------------------------------------------
```

```
 Atomic number (AN) and Cartesian coordinates
  AN         X            Y            Z
 ---------------------------------------------------
  O     -2.127091      0.458233      1.245334
  C     -1.348538      1.187501      0.379961
  H     -0.419856      1.574812      0.790135
  C     -1.950465      1.257367     -0.860438
  H     -1.545781      1.763475     -1.734073
  C     -3.178018      0.517232     -0.754435
  H     -3.924935      0.345478     -1.527173
  C     -3.235151      0.051453      0.545610
  H     -3.958529     -0.538935      1.101699
  O      1.370740      1.741153     -1.161649
  C      1.598175      2.794360     -0.312583
  H      1.353174      3.779555     -0.700343
  C      2.118798      2.340830      0.884892
  H      2.394105      2.960303      1.736195
  C      2.213265      0.911865      0.763176
  H      2.568170      0.197231      1.502207
  C      1.742208      0.598955     -0.496115
  H      1.605593     -0.333832     -1.034741
  O      0.292011     -2.404284     -1.185908
  C      1.447961     -2.866385     -0.606662
  H      2.170247     -3.317628     -1.281795
  C      1.414372     -2.659561      0.759352
  H      2.196664     -2.934933      1.464433
  C      0.155480     -2.025605      1.037720
  H     -0.231822     -1.692994      1.998320
  C     -0.486804     -1.890680     -0.176320
  H     -1.440012     -1.467344     -0.482019
 ---------------------------------------------------

Cartesian coordinates of :  Furan3_2

 ---------------------------------------------------
 Atomic number (AN) and Cartesian coordinates
  AN         X            Y            Z
 ---------------------------------------------------
  C      1.567801      1.732163      1.293385
  C      1.810034      2.786713      0.347926
  C      2.103085      2.165391     -0.851702
  C      1.726809      0.547348      0.602223
  O      2.059765      0.802118     -0.705512
  H      1.292467      1.826252      2.341583
  H      1.771285      3.860824      0.518404
  H      1.642095     -0.501452      0.872706
  H      2.361677      2.529139     -1.842562
  C     -3.388273      0.251172     -0.457301
  C     -2.307467      0.703394     -1.289562
  C     -1.350414      1.218339     -0.437393
  C     -3.010699      0.529417      0.843381
  O     -1.774418      1.121450      0.864899
  H     -4.320779     -0.217278     -0.766040
  H     -2.230859      0.646875     -2.373312
```

H	-3.486489	0.386355	1.809995
H	-0.376987	1.676906	-0.584906
C	0.141403	-2.700085	1.059947
C	1.383885	-3.052043	0.427739
C	1.362410	-2.450476	-0.816680
C	-0.542211	-1.908177	0.157041
O	0.193784	-1.757291	-0.990887
H	-0.207522	-2.985233	2.050499
H	2.189775	-3.663565	0.828876
H	-1.508825	-1.412060	0.169238
H	2.062936	-2.415921	-1.646504

Cartesian coordinates of : Furan3_3

Atomic number (AN) and Cartesian coordinates

AN	X	Y	Z
C	-0.116734	-2.722422	1.130378
C	0.691633	-1.880036	0.293293
C	0.028329	-1.791140	-0.914079
C	-1.214284	-3.084322	0.371335
O	-1.132978	-2.523779	-0.878593
H	0.075563	-3.025636	2.157958
H	1.626663	-1.383418	0.544233
H	-2.083133	-3.710963	0.554484
H	0.223304	-1.256810	-1.838952
C	-2.071531	1.539976	1.249837
C	-2.145353	2.649994	0.339363
C	-2.020061	2.113239	-0.928028
C	-1.902971	0.410604	0.472942
O	-1.876159	0.750791	-0.856561
H	-2.129859	1.563533	2.336193
H	-2.272230	3.704232	0.577270
H	-1.789355	-0.645991	0.699455
H	-2.023831	2.538639	-1.928028
C	3.364096	0.663862	0.589217
C	2.190127	1.328773	1.085826
C	1.305019	1.395384	0.028821
C	3.108122	0.376102	-0.737575
O	1.859254	0.821882	-1.089445
H	4.278362	0.426184	1.129548
H	2.010949	1.705654	2.090688
H	3.680098	-0.106929	-1.525290
H	0.304183	1.800273	-0.088740

Cartesian coordinates of : Furan3_4

Atomic number (AN) and Cartesian coordinates

AN	X	Y	Z

C	1.422402	-1.239354	-0.582082
C	1.629460	-1.735184	0.749687
C	0.897661	-2.903056	0.846608
C	0.581979	-2.141774	-1.200431
O	0.253106	-3.161947	-0.339189
H	1.830614	-0.331606	-1.020448
H	2.232977	-1.287822	1.536712
H	0.130119	-2.194333	-2.186918
H	0.746683	-3.635207	1.635472
C	-3.174891	1.243927	0.408959
C	-2.430840	0.388482	1.292148
C	-1.767780	-0.517987	0.488185
C	-2.913967	0.790759	-0.870460
O	-2.062381	-0.283994	-0.831396
H	-3.817194	2.082123	0.671793
H	-2.383035	0.432126	2.378512
H	-3.247711	1.097093	-1.858311
H	-1.083086	-1.336705	0.688067
C	2.441954	1.840583	0.794067
C	1.049222	1.678575	1.105403
C	0.362913	1.863438	-0.077866
C	2.503534	2.112700	-0.559601
O	1.241628	2.134556	-1.098572
H	3.293249	1.769053	1.468656
H	0.599678	1.445416	2.068036
H	3.318366	2.317910	-1.248871
H	-0.689373	1.846375	-0.347145

Cartesian coordinates of : Furan3_5

Atomic number (AN) and Cartesian coordinates

AN	X	Y	Z
C	3.412619	-0.318815	-0.456912
C	2.328684	-0.828167	-1.251162
C	1.359756	-1.250077	-0.361332
C	3.024619	-0.473878	0.860710
O	1.779571	-1.043437	0.928095
H	4.354015	0.104948	-0.801184
H	2.265188	-0.880857	-2.336422
H	3.498629	-0.252760	1.813380
H	0.382895	-1.711957	-0.471752
C	-0.135508	2.733491	1.019877
C	0.621404	1.955353	0.079109
C	-0.191664	1.794989	-1.024704
C	-1.354268	2.989657	0.419308
O	-1.396196	2.424853	-0.830926
H	0.167907	3.062672	2.012033
H	1.623787	1.548482	0.195046
H	-2.242143	3.538865	0.721270
H	-0.077833	1.274196	-1.970675
C	-1.848731	-2.775337	0.377460

C	-1.652556	-1.694383	1.304077
C	-1.763975	-0.529628	0.570981
C	-2.069511	-2.188096	-0.854098
O	-2.023819	-0.820995	-0.745619
H	-1.828810	-3.844033	0.581555
H	-1.441129	-1.759068	2.369236
H	-2.278282	-2.579256	-1.846302
H	-1.685889	0.524757	0.821528

Cartesian coordinates of : Furan3_6

Atomic number (AN) and Cartesian coordinates

AN	X	Y	Z
C	-2.218639	1.495349	1.171616
C	-3.269028	0.974043	0.339860
C	-2.713464	0.804818	-0.914839
C	-1.102287	1.601976	0.364203
O	-1.398469	1.188801	-0.909504
H	-2.269663	1.753906	2.227435
H	-4.296534	0.750289	0.619922
H	-0.081616	1.936644	0.528199
H	-3.098243	0.442759	-1.864348
C	0.790993	-3.318038	0.339834
C	-0.185615	-2.669021	1.171644
C	-0.836212	-1.755584	0.364253
C	0.659658	-2.752335	-0.914873
O	-0.330399	-1.805528	-0.909492
H	1.498566	-4.095978	0.619868
H	-0.383926	-2.842448	2.227487
H	1.165520	-2.904561	-1.864420
H	-1.636387	-1.039009	0.528292
C	2.404357	1.173705	1.171590
C	2.478064	2.344026	0.339838
C	2.053726	1.947505	-0.914860
C	1.938529	0.153599	0.364176
O	1.728787	0.616689	-0.909528
H	2.653793	1.088613	2.227407
H	2.798024	3.345754	0.619903
H	1.718065	-0.897672	0.528169
H	1.932554	2.461763	-1.864367

Cartesian coordinates of : Furan3_7

Atomic number (AN) and Cartesian coordinates

AN	X	Y	Z
C	-0.298417	-1.962516	0.256745
C	0.332525	-1.922514	-1.032769
C	1.610211	-2.413943	-0.854832

C	0.641473	-2.476935	1.126913
O	1.812613	-2.756115	0.461671
H	-1.306147	-1.642907	0.513911
H	-0.086738	-1.552318	-1.965473
H	0.640668	-2.698381	2.190832
H	2.455207	-2.574193	-1.518981
C	-2.564339	0.688411	1.199490
C	-3.462945	-0.089193	0.390741
C	-2.929234	-0.084062	-0.884039
C	-1.549306	1.105801	0.361052
O	-1.768600	0.644935	-0.913120
H	-2.645343	0.910140	2.261757
H	-4.379981	-0.588058	0.698173
H	-0.654868	1.705762	0.502476
H	-3.242199	-0.518311	-1.829914
C	1.594735	3.020649	0.308069
C	1.943309	1.904678	1.144756
C	1.825980	0.776805	0.355413
C	1.293940	2.488313	-0.931619
O	1.435708	1.125285	-0.912770
H	1.564894	4.074934	0.576008
H	2.237307	1.922314	2.192344
H	0.980915	2.919595	-1.878611
H	1.970934	-0.284391	0.531698

Cartesian coordinates of : Furan3_8

Atomic number (AN) and Cartesian coordinates

AN	X	Y	Z
O	2.267790	0.885344	-1.158893
C	2.583002	2.042183	-0.490757
H	2.888098	2.880810	-1.110904
C	2.450776	1.858354	0.873074
H	2.639233	2.609163	1.638243
C	2.021844	0.499324	1.052872
H	1.802563	-0.017656	1.984604
C	1.922341	-0.046500	-0.211248
H	1.638775	-1.025866	-0.584836
O	-1.900594	1.521240	-1.158889
C	-3.060074	1.215851	-0.490768
H	-3.938885	1.060763	-1.110929
C	-2.834793	1.193305	0.873069
H	-3.579263	0.981155	1.638229
C	-1.443369	1.501329	1.052886
H	-0.886030	1.569940	1.984628
C	-0.920888	1.688008	-0.211229
H	0.069065	1.932069	-0.584806
O	-0.367267	-2.406596	-1.158849
C	0.477058	-3.257998	-0.490813
H	1.050745	-3.941499	-1.111032
C	0.384082	-3.051619	0.873033

```
 H      0.940153     -3.590242      1.638136
 C     -0.578419     -2.000669      1.052946
 H     -0.916431     -1.552317      1.984721
 C     -1.001482     -1.641555     -0.211126
 H     -1.707915     -0.906311     -0.584629
---------------------------------------------------
```

Cartesian coordinates of : Furan3_9

```
---------------------------------------------------
Atomic number (AN) and Cartesian coordinates
 AN          X            Y            Z
---------------------------------------------------
 C      0.718783     -1.899457     -0.091015
 C     -0.054618     -1.967339      1.116923
 C     -1.215602     -2.642745      0.797894
 C     -0.027814     -2.539319     -1.059702
 O     -1.212364     -2.997070     -0.530662
 H      1.691536     -1.432318     -0.230662
 H      0.200236     -1.557193      2.091601
 H      0.127405     -2.747278     -2.115013
 H     -2.098132     -2.933027      1.361476
 C     -1.793671      1.570450     -1.278912
 C     -1.992448      2.683037     -0.391442
 C     -2.151735      2.142019      0.870449
 C     -1.842124      0.435004     -0.494588
 O     -2.065279      0.773667      0.817391
 H     -1.620734      1.596281     -2.352622
 H     -2.012038      3.742320     -0.639754
 H     -1.737089     -0.625229     -0.700056
 H     -2.336212      2.568566      1.852821
 C      2.256748      0.923954      1.249782
 C      3.390987      0.493715      0.479673
 C      3.030308      0.643782     -0.846391
 C      1.287857      1.297673      0.339001
 O      1.754389      1.136196     -0.941119
 H      2.158933      0.954139      2.333212
 H      4.347393      0.124001      0.844298
 H      0.281939      1.696764      0.427945
 H      3.542764      0.465980     -1.788156
---------------------------------------------------
```

Cartesian coordinates of : Furan3_10

```
---------------------------------------------------
Atomic number (AN) and Cartesian coordinates
 AN          X            Y            Z
---------------------------------------------------
 C     -0.051216      1.487302      0.321493
 C     -1.045202      2.283398      0.986831
 C     -2.134598      2.329005      0.138320
 C     -0.609461      1.110011     -0.883400
 O     -1.876145      1.622547     -1.007339
 H      0.943064      1.224750      0.676206
```

H	-0.974849	2.763123	1.961451
H	-0.265003	0.498758	-1.711504
H	-3.109071	2.807260	0.191929
C	3.317808	-0.004511	0.998609
C	3.565982	0.770306	-0.186819
C	2.755789	0.236166	-1.168551
C	2.374106	-0.951095	0.651634
O	2.027531	-0.814409	-0.669426
H	3.768809	0.116464	1.981456
H	4.243641	1.613219	-0.305445
H	1.855925	-1.735059	1.195180
H	2.590350	0.474109	-2.215821
C	-2.723320	-1.067546	0.301947
C	-2.250235	-1.781458	-0.851623
C	-0.978010	-2.218239	-0.539680
C	-1.703608	-1.120611	1.230045
O	-0.639412	-1.829671	0.731539
H	-3.680464	-0.567746	0.431660
H	-2.770533	-1.948146	-1.792783
H	-1.583917	-0.728729	2.235785
H	-0.221949	-2.782110	-1.079136

Cartesian coordinates of : Furan3_11

Atomic number (AN) and Cartesian coordinates

AN	X	Y	Z
C	-0.725192	2.194846	0.969466
C	-2.135852	2.322915	0.726774
C	-2.324188	2.016323	-0.605936
C	-0.158290	1.821873	-0.231939
O	-1.125226	1.717187	-1.201353
H	-0.188774	2.352206	1.903093
H	-2.914961	2.601416	1.433992
H	0.860756	1.620567	-0.549445
H	-3.199189	1.968069	-1.248437
C	-0.787870	-2.863205	-0.740784
C	-1.105492	-3.043120	0.649595
C	-1.554220	-1.814678	1.100926
C	-1.065035	-1.540344	-1.030117
O	-1.537524	-0.899361	0.084114
H	-0.396829	-3.601773	-1.437529
H	-1.017605	-3.950710	1.243580
H	-0.975831	-0.926120	-1.921587
H	-1.911339	-1.449912	2.060380
C	2.454495	0.123562	1.268230
C	3.281953	0.883837	0.373144
C	3.008197	0.409327	-0.896023
C	1.733770	-0.753792	0.481840
O	2.070834	-0.590763	-0.839208
H	2.387761	0.210753	2.350870
H	3.986186	1.675779	0.620845

H	0.987243	-1.514849	0.690934
H	3.384260	0.652812	-1.886170

Cartesian coordinates of : Furan3_12

Atomic number (AN) and Cartesian coordinates

AN	X	Y	Z
C	-2.997497	0.062371	0.168540
C	-3.765912	-1.122291	-0.102411
C	-2.864741	-2.167422	-0.120840
C	-1.685129	-0.344427	0.297052
O	-1.594866	-1.706661	0.122567
H	-3.354748	1.085664	0.259641
H	-4.839102	-1.201596	-0.265979
H	-0.750651	0.167234	0.509738
H	-2.958329	-3.237821	-0.284630
C	1.558462	-1.680345	0.686628
C	2.902660	-1.333297	1.055031
C	3.508261	-0.861544	-0.094713
C	1.442374	-1.392633	-0.659266
O	2.626406	-0.897127	-1.145428
H	0.769464	-2.082593	1.317619
H	3.371222	-1.418186	2.033805
H	0.630440	-1.472836	-1.375710
H	4.506681	-0.499254	-0.325305
C	0.434816	2.419453	1.140022
C	-0.713088	2.965185	0.468500
C	-0.611262	2.563708	-0.849228
C	1.150659	1.726638	0.182671
O	0.521768	1.814223	-1.034339
H	0.703106	2.517534	2.189934
H	-1.510707	3.571500	0.893500
H	2.075830	1.157018	0.204114
H	-1.227288	2.717473	-1.731029

Cartesian coordinates of : Furan4_ 1

Atomic number (AN) and Cartesian coordinates

AN	X	Y	Z
O	-0.775679	-1.623276	-0.835198
C	-0.214132	-2.739150	-0.268869
H	0.823330	-2.927611	-0.532039
C	-1.139065	-3.385791	0.528767
H	-0.967288	-4.298619	1.095899
C	-2.347010	-2.608604	0.450530

H	-3.296609	-2.800124	0.946392
C	-2.069527	-1.545389	-0.387402
H	-2.645507	-0.695323	-0.745133
O	-0.521173	0.750292	2.691316
C	-0.026424	-0.371191	2.068617
H	-0.717957	-1.202807	1.968069
C	1.280742	-0.162508	1.676063
H	1.915370	-0.873263	1.152558
C	1.613121	1.176058	2.078142
H	2.556452	1.697772	1.933326
C	0.483169	1.685868	2.688720
H	0.245245	2.638697	3.154121
O	2.512739	-1.037902	-1.316798
C	1.745513	0.056105	-1.641636
H	0.764920	-0.156432	-2.053924
C	2.421230	1.219495	-1.335067
H	2.047573	2.229664	-1.490449
C	3.684413	0.818943	-0.778637
H	4.491814	1.455830	-0.422053
C	3.688200	-0.562144	-0.789360
H	4.414267	-1.313561	-0.490297
O	-1.187974	1.353172	-1.675799
C	-2.497098	1.563773	-2.029154
H	-2.734061	1.429085	-3.081234
C	-3.238566	1.927493	-0.920651
H	-4.302707	2.156094	-0.912093
C	-2.322912	1.938099	0.187080
H	-2.535771	2.158905	1.230480
C	-1.091177	1.577811	-0.322469
H	-0.105231	1.430201	0.110164

Cartesian coordinates of : Furan4_ 2

Atomic number (AN) and Cartesian coordinates

AN	X	Y	Z
C	1.518524	3.085628	0.362497
C	2.084292	1.766936	0.446669
C	1.391104	1.112688	1.445630
C	0.520667	3.139007	1.316674
O	0.437136	1.943161	1.985727
H	1.799459	3.892217	-0.311726
H	2.875737	1.333856	-0.161625
H	-0.172966	3.912023	1.636653
H	1.443988	0.111744	1.864121
C	1.560040	-0.668934	-2.141724
C	2.995589	-0.695222	-2.077540
C	3.314561	-1.241254	-0.848627
C	1.108368	-1.200100	-0.949944
O	2.172466	-1.555116	-0.155723
H	0.929679	-0.305857	-2.950270
H	3.706352	-0.362653	-2.831953

H	0.120445	-1.392285	-0.541844
H	4.252109	-1.470652	-0.348783
C	-3.482409	1.131820	-1.101554
C	-2.400359	1.774729	-0.408044
C	-1.236094	1.212396	-0.894465
C	-2.897363	0.223889	-1.963929
O	-1.532203	0.268519	-1.847853
H	-4.550219	1.310972	-0.989775
H	-2.461414	2.553874	0.348969
H	-3.290427	-0.479015	-2.693808
H	-0.179669	1.361186	-0.688960
C	-2.113332	-1.651975	0.801741
C	-1.556912	-2.975553	0.726087
C	-0.418352	-2.961896	1.508499
C	-1.273060	-0.928355	1.624937
O	-0.239678	-1.720489	2.066074
H	-2.998994	-1.262390	0.304822
H	-1.933513	-3.827956	0.164255
H	-1.271887	0.096893	1.982412
H	0.337970	-3.703384	1.750263

Cartesian coordinates of : Furan4_ 3

Atomic number (AN) and Cartesian coordinates

AN	X	Y	Z
C	-3.289898	1.563352	0.861027
C	-2.115900	2.212437	0.344302
C	-1.034231	1.570409	0.916219
C	-2.836751	0.574005	1.713391
O	-1.466692	0.575521	1.758625
H	-4.331520	1.789280	0.640505
H	-2.059845	3.036560	-0.363652
H	-3.332253	-0.161881	2.341071
H	0.043300	1.680301	0.826456
C	-2.271534	-2.207245	-0.423856
C	-1.437959	-3.304479	-0.834253
C	-0.450781	-2.758387	-1.631068
C	-1.730665	-1.072612	-0.997377
O	-0.623085	-1.402165	-1.740646
H	-3.153893	-2.238068	0.211519
H	-1.540922	-4.356436	-0.576012
H	-1.987363	-0.017424	-0.962160
H	0.405270	-3.172294	-2.156351
C	2.269161	2.008529	-0.452714
C	1.682230	0.926744	-1.193668
C	0.620172	1.470016	-1.887557
C	1.520631	3.131869	-0.749086
O	0.513207	2.815626	-1.627023
H	3.125326	1.964991	0.216611
H	1.983650	-0.117475	-1.200558
H	1.571988	4.173144	-0.442236

H	-0.115898	1.059306	-2.571978
C	1.537170	-0.535672	1.988478
C	2.960018	-0.601354	1.800670
C	3.171784	-1.519463	0.790286
C	0.983512	-1.414763	1.078822
O	1.975157	-2.026052	0.347845
H	0.981221	0.073201	2.697698
H	3.733008	-0.055137	2.338290
H	-0.034785	-1.709285	0.842072
H	4.062276	-1.912548	0.306626

Cartesian coordinates of : Furan4_ 4

Atomic number (AN) and Cartesian coordinates

AN	X	Y	Z
C	-2.487749	-1.700271	1.787868
C	-1.159146	-1.204929	2.020769
C	-0.353408	-1.788830	1.063700
C	-2.394711	-2.555235	0.706255
O	-1.097696	-2.621116	0.261089
H	-3.395822	-1.469870	2.342175
H	-0.827259	-0.506040	2.785103
H	-3.112933	-3.169372	0.169226
H	0.704332	-1.726618	0.829532
C	1.084536	2.873058	0.565118
C	2.453934	2.631428	0.930886
C	2.460197	1.435043	1.621488
C	0.356476	1.806114	1.055961
O	1.189163	0.928317	1.707481
H	0.677770	3.717509	0.013505
H	3.323675	3.248925	0.715279
H	-0.691720	1.523800	1.010985
H	3.241976	0.836972	2.081053
C	-2.628355	2.477470	-0.804934
C	-2.835115	1.119609	-0.381345
C	-1.869608	0.363603	-1.017693
C	-1.553125	2.447980	-1.672819
O	-1.089026	1.166053	-1.813169
H	-3.192075	3.362317	-0.515339
H	-3.584750	0.737356	0.307449
H	-1.035985	3.206462	-2.254424
H	-1.606198	-0.689456	-1.003332
C	1.632190	-2.468247	-1.755843
C	1.124939	-1.146200	-1.996923
C	1.796064	-0.306572	-1.129574
C	2.581098	-2.339713	-0.760352
O	2.692375	-1.025807	-0.373748
H	1.335481	-3.397240	-2.238319
H	0.364239	-0.839694	-2.710987
H	3.234028	-3.041668	-0.248464
H	1.757420	0.761195	-0.932032

Cartesian coordinates of : Furan4_ 5

Atomic number (AN) and Cartesian coordinates

AN	X	Y	Z
O	-0.689904	-1.854826	-0.955360
C	-0.065549	-2.951391	-0.415336
H	0.869322	-3.242839	-0.886191
C	-0.799022	-3.442121	0.648048
H	-0.539480	-4.304580	1.258792
C	-1.946870	-2.585119	0.772182
H	-2.748135	-2.645430	1.505565
C	-1.830182	-1.633719	-0.221395
H	-2.434255	-0.783265	-0.526453
O	-0.752026	0.236050	2.415076
C	0.387304	-0.227224	1.803277
H	0.351272	-1.252835	1.445776
C	1.357962	0.756590	1.793410
H	2.353577	0.670206	1.362754
C	0.773516	1.903959	2.430367
H	1.229534	2.876360	2.606808
C	-0.510015	1.532244	2.786869
H	-1.323797	2.040183	3.297914
O	2.559932	-0.916640	-0.747420
C	1.841710	0.058911	-1.397206
H	0.834373	-0.214106	-1.694398
C	2.598195	1.207594	-1.515071
H	2.276462	2.133766	-1.987283
C	3.862733	0.924897	-0.893020
H	4.720738	1.586299	-0.788181
C	3.785518	-0.379607	-0.443382
H	4.478873	-1.037846	0.073642
O	-1.160097	1.138003	-1.803064
C	-2.454227	1.138521	-2.257423
H	-2.602071	0.782780	-3.273562
C	-3.303942	1.620670	-1.279656
H	-4.382803	1.731296	-1.369940
C	-2.474139	1.933675	-0.148633
H	-2.781644	2.330613	0.816068
C	-1.180421	1.617932	-0.514540
H	-0.220634	1.653824	-0.006104

Cartesian coordinates of : Furan4_ 6

Atomic number (AN) and Cartesian coordinates

AN	X	Y	Z
C	1.046836	-0.156209	2.961900
C	-0.316431	-0.612169	2.977692

C	-0.497613	-1.328860	1.810017
C	1.598509	-0.629425	1.785589
O	0.665922	-1.351028	1.083743
H	1.559225	0.444889	3.710587
H	-1.070257	-0.436566	3.742838
H	2.580004	-0.545177	1.325131
H	-1.341128	-1.847294	1.361521
C	-3.121890	-1.853355	-0.705059
C	-2.183388	-2.917696	-0.945442
C	-1.004698	-2.310146	-1.331724
C	-2.446295	-0.676032	-0.960950
O	-1.158675	-0.947410	-1.350245
H	-4.158332	-1.938115	-0.384093
H	-2.347912	-3.988952	-0.847304
H	-2.717209	0.375921	-0.916559
H	-0.017070	-2.677858	-1.597260
C	-1.840875	2.132286	1.101102
C	-2.588385	2.850287	0.105526
C	-1.833407	2.799650	-1.051591
C	-0.686710	1.695140	0.482329
O	-0.671902	2.103630	-0.830498
H	-2.113621	1.944317	2.137107
H	-3.553634	3.340906	0.216316
H	0.179283	1.128432	0.811771
H	-1.968718	3.193210	-2.055630
C	2.624039	1.387477	-0.905835
C	3.935793	0.879532	-0.611825
C	3.887782	-0.478324	-0.867136
C	1.871967	0.304491	-1.316148
O	2.635817	-0.837282	-1.298233
H	2.266676	2.411719	-0.823811
H	4.805341	1.432402	-0.261094
H	0.837066	0.178966	-1.618244
H	4.619574	-1.279970	-0.810088

Cartesian coordinates of : Furan4_ 7

Atomic number (AN) and Cartesian coordinates

AN	X	Y	Z
O	-1.194895	1.257190	1.734622
C	-2.500575	1.405545	2.128519
H	-2.709883	1.193284	3.173611
C	-3.277458	1.819479	1.062934
H	-4.347391	2.016756	1.092266
C	-2.387890	1.928933	-0.060738
H	-2.629338	2.218086	-1.080629
C	-1.135216	1.571776	0.397440
H	-0.158847	1.469166	-0.065347
O	1.352721	1.345148	-1.941774
C	0.273904	1.760509	-2.683098
H	0.288596	2.800418	-2.998812

```
 C     -0.610331      0.715155     -2.870226
 H     -1.548656      0.762651     -3.419869
 C     -0.041185     -0.418586     -2.196777
 H     -0.457250     -1.418848     -2.099522
 C      1.148696      0.016482     -1.648497
 H      1.928839     -0.458112     -1.060413
 O     -0.652311     -1.626533      0.757672
 C     -1.947731     -1.605282      0.309064
 H     -2.542728     -0.748186      0.614535
 C     -2.204061     -2.726410     -0.456888
 H     -3.150540     -2.970275     -0.935529
 C     -0.980447     -3.481325     -0.488058
 H     -0.790277     -4.425964     -0.993968
 C     -0.068475     -2.765014      0.263924
 H      0.972540     -2.915083      0.537431
 O      2.574260     -0.980644      1.312789
 C      1.788192      0.096190      1.650261
 H      0.838939     -0.137276      2.120697
 C      2.402300      1.271857      1.268837
 H      2.000733      2.273258      1.409316
 C      3.644949      0.898446      0.651875
 H      4.398719      1.551963      0.217611
 C      3.700863     -0.480621      0.704143
 H      4.435141     -1.215932      0.385854
 ---------------------------------------------------
```

```
Cartesian coordinates of :  Furan4_ 8

 ---------------------------------------------------
 Atomic number (AN) and Cartesian coordinates
  AN          X            Y            Z
 ---------------------------------------------------
 C      2.266081     -1.873310     -0.114972
 C      2.875433     -1.705582     -1.405678
 C      1.839827     -1.664326     -2.319340
 C      0.904463     -1.919754     -0.332441
 O      0.634310     -1.799977     -1.675926
 H      2.753053     -1.933051      0.855989
 H      3.935409     -1.627871     -1.641181
 H      0.039577     -2.013124      0.319385
 H      1.792982     -1.563717     -3.400419
 C     -3.685014      0.953793     -0.739256
 C     -3.600351     -0.480580     -0.774222
 C     -2.348097     -0.780743     -1.273698
 C     -2.477793      1.421569     -1.222199
 O     -1.659264      0.372083     -1.553452
 H     -4.520188      1.566388     -0.405178
 H     -4.357970     -1.201670     -0.473550
 H     -2.060791      2.411836     -1.379527
 H     -1.825343     -1.705842     -1.496862
 C     -1.143028      0.038804      1.701523
 C     -1.578678     -1.238142      2.195818
 C     -0.478835     -1.814230      2.803763
 C      0.190034      0.148586      2.043235
```

O	0.604889	-0.975665	2.717834
H	-1.726946	0.772841	1.150606
H	-2.569869	-1.679581	2.113800
H	0.949542	0.910071	1.887646
H	-0.310897	-2.755359	3.320799
C	2.406328	2.787970	0.630844
C	2.533754	1.656462	-0.246029
C	1.271801	1.407960	-0.747805
C	1.072949	3.150529	0.597399
O	0.375443	2.320164	-0.243049
H	3.185499	3.274809	1.214192
H	3.428758	1.083279	-0.475310
H	0.494980	3.933825	1.080775
H	0.855947	0.660275	-1.416081

Cartesian coordinates of : Furan4_ 9

Atomic number (AN) and Cartesian coordinates

AN	X	Y	Z
C	-2.987745	0.684776	-2.108425
C	-1.550856	0.680440	-2.091399
C	-1.176612	1.168944	-0.855152
C	-3.384738	1.177633	-0.879259
O	-2.288804	1.478100	-0.110015
H	-3.649215	0.372043	-2.914272
H	-0.869535	0.353988	-2.873692
H	-4.352643	1.379406	-0.427682
H	-0.219560	1.331861	-0.370427
C	2.433626	-1.733692	-0.255526
C	3.497171	-1.197558	-1.060093
C	2.892635	-0.390105	-2.005444
C	1.259832	-1.214116	-0.765330
O	1.531536	-0.397819	-1.836659
H	2.511071	-2.410624	0.592432
H	4.566192	-1.378390	-0.964593
H	0.211660	-1.301385	-0.497977
H	3.267215	0.213763	-2.827797
C	-1.376833	-1.175102	1.678574
C	-0.494013	-2.223641	2.109537
C	-0.617071	-3.237828	1.178370
C	-1.975430	-1.626699	0.518791
O	-1.520074	-2.886116	0.207948
H	-1.545266	-0.203856	2.137658
H	0.145009	-2.242028	2.990749
H	-2.692095	-1.195384	-0.175995
H	-0.165260	-4.220300	1.071587
C	1.238835	1.099558	1.808336
C	0.361356	2.179337	2.166011
C	0.621510	3.198409	1.269800
C	1.971301	1.538517	0.722321
O	1.603970	2.820591	0.389721

```
 H      1.317995      0.116654      2.266157
 H     -0.372505      2.211214      2.968916
 H      2.741962      1.086312      0.103196
 H      0.214132      4.195435      1.127104
---------------------------------------------------
```

Cartesian coordinates of : Furan4_10

```
---------------------------------------------------
Atomic number (AN) and Cartesian coordinates
 AN         X            Y             Z
---------------------------------------------------
 C     -1.841001     -0.290766     -2.123045
 C     -3.253191     -0.094947     -1.942748
 C     -3.560893     -0.647772     -0.714516
 C     -1.390220     -0.948220     -0.995214
 O     -2.435111     -1.172906     -0.132292
 H     -1.233526      0.017256     -2.971110
 H     -3.955385      0.384343     -2.622367
 H     -0.416887     -1.306759     -0.671237
 H     -4.482440     -0.753342     -0.147992
 C     -1.745077      2.085880      0.562140
 C     -0.929532      3.266538      0.473416
 C      0.129128      3.070967      1.339429
 C     -1.125017      1.257354      1.476583
 O      0.017013      1.851823      1.959046
 H     -2.653626      1.851972      0.011140
 H     -1.089576      4.144522     -0.149188
 H     -1.350568      0.270222      1.869667
 H      1.000362      3.661467      1.608729
 C      1.841097      0.290795     -2.122964
 C      3.253281      0.094990     -1.942601
 C      3.560916      0.647804     -0.714347
 C      1.390252      0.948222     -0.995141
 O      2.435098      1.172909     -0.132166
 H      1.233667     -0.017217     -2.971063
 H      3.955514     -0.384277     -2.622195
 H      0.416898      1.306744     -0.671206
 H      4.482435      0.753381     -0.147779
 C      1.745057     -2.085883      0.562183
 C      0.929520     -3.266543      0.473395
 C     -0.129172     -3.070999      1.339375
 C      1.124951     -1.257379      1.476616
 O     -0.017092     -1.851868      1.959022
 H      2.653625     -1.851953      0.011227
 H      1.089594     -4.144512     -0.149224
 H      1.350476     -0.270252      1.869725
 H     -1.000411     -3.661512      1.608630
---------------------------------------------------
```

Cartesian coordinates of : Furan4_11

```
---------------------------------------------------
Atomic number (AN) and Cartesian coordinates
```

AN	X	Y	Z
C	-2.375333	-1.766760	0.316711
C	-3.467259	-1.206917	1.064574
C	-2.897093	-0.353172	1.990661
C	-1.221794	-1.214276	0.840505
O	-1.532835	-0.354853	1.863925
H	-2.420691	-2.485002	-0.499108
H	-4.531126	-1.404337	0.946408
H	-0.164359	-1.317789	0.618917
H	-3.301033	0.279942	2.776334
C	-2.092276	1.665347	-0.686273
C	-1.610344	3.018394	-0.633849
C	-0.513189	3.069989	-1.472367
C	-1.251668	0.992321	-1.550738
O	-0.288793	1.840678	-2.040605
H	-2.927966	1.223516	-0.147506
H	-2.008641	3.848431	-0.053696
H	-1.199014	-0.036352	-1.890878
H	0.172147	3.861055	-1.764857
C	1.273574	-1.171495	-1.886556
C	0.446504	-2.318998	-2.143171
C	0.688856	-3.206746	-1.111626
C	1.958396	-1.442517	-0.718790
O	1.612884	-2.685749	-0.242354
H	1.351785	-0.255029	-2.467783
H	-0.231908	-2.482742	-2.978924
H	2.685123	-0.891733	-0.127255
H	0.313955	-4.197077	-0.867667
C	3.408905	1.269762	1.132867
C	2.324982	1.847143	0.386773
C	1.165983	1.279896	0.881179
C	2.829677	0.390995	2.030080
O	1.466691	0.394373	1.886766
H	4.473952	1.469786	1.031449
H	2.381584	2.583726	-0.411582
H	3.225145	-0.265295	2.800908
H	0.109934	1.395517	0.653506

Cartesian coordinates of : Furan4_12

Atomic number (AN) and Cartesian coordinates

AN	X	Y	Z
C	1.434045	-2.659664	0.533285
C	0.380302	-3.540068	0.957324
C	-0.459848	-2.775497	1.744558
C	1.157365	-1.424007	1.088258
O	0.006685	-1.490909	1.834328
H	2.290532	-2.895491	-0.093796
H	0.253806	-4.595356	0.722707
H	1.639704	-0.451537	1.044295

H	-1.368435	-2.990746	2.300736
C	3.540084	0.380262	-0.957363
C	2.659706	1.434001	-0.533266
C	1.424030	1.157354	-1.088211
C	2.775481	-0.459854	-1.744600
O	1.490896	0.006695	-1.834318
H	4.595376	0.253743	-0.722771
H	2.895559	2.290460	0.093845
H	2.990703	-1.368427	-2.300812
H	0.451563	1.639696	-1.044190
C	-2.659725	-1.434017	-0.533243
C	-3.540100	-0.380280	-0.957360
C	-2.775484	0.459830	-1.744592
C	-1.424042	-1.157374	-1.088174
O	-1.490900	-0.006722	-1.834290
H	-2.895589	-2.290473	0.093868
H	-4.595394	-0.253759	-0.722782
H	-0.451574	-1.639714	-1.044140
H	-2.990699	1.368402	-2.300807
C	-0.380312	3.540092	0.957308
C	-1.434040	2.659671	0.533270
C	-1.157339	1.424017	1.088237
C	0.459853	2.775533	1.744535
O	-0.006658	1.490938	1.834303
H	-0.253837	4.595384	0.722696
H	-2.290534	2.895483	-0.093807
H	1.368436	2.990798	2.300714
H	-1.639651	0.451535	1.044255

Cartesian coordinates of : Furan4_13

Atomic number (AN) and Cartesian coordinates

AN	X	Y	Z
C	3.970152	0.525460	-0.623748
C	2.802638	1.330575	-0.856939
C	1.814308	0.467028	-1.289021
C	3.605815	-0.772003	-0.931579
O	2.299241	-0.815765	-1.342646
H	4.949488	0.849635	-0.277035
H	2.697645	2.405006	-0.720238
H	4.134268	-1.721649	-0.923127
H	0.776194	0.590724	-1.581101
C	-2.117775	-2.261628	-0.244979
C	-2.974015	-1.978055	-1.364241
C	-2.192157	-1.303020	-2.283102
C	-0.878367	-1.739467	-0.559952
O	-0.915761	-1.157748	-1.804402
H	-2.373191	-2.778121	0.677604
H	-4.024942	-2.233391	-1.485579
H	0.076776	-1.695591	-0.043960
H	-2.380320	-0.888394	-3.269833

C	-2.271254	2.221171	0.953820
C	-1.336166	3.299350	0.782774
C	-0.653651	3.032250	-0.389144
C	-2.089528	1.374314	-0.121249
O	-1.110915	1.867282	-0.950864
H	-2.972994	2.070277	1.771188
H	-1.180965	4.159621	1.430944
H	-2.541444	0.434671	-0.428062
H	0.124084	3.552622	-0.942164
C	1.089077	-1.782341	2.426442
C	1.505052	-0.559258	1.798355
C	0.393759	0.262353	1.780211
C	-0.245000	-1.613366	2.749499
O	-0.674752	-0.369620	2.367595
H	1.684822	-2.672976	2.617720
H	2.484978	-0.314552	1.393904
H	-0.984065	-2.241983	3.239369
H	0.209823	1.270737	1.420022

Cartesian coordinates of : Furan4_14

Atomic number (AN) and Cartesian coordinates

AN	X	Y	Z
C	3.066952	-0.969754	-1.468479
C	4.176936	-0.275311	-0.874123
C	3.688696	0.953461	-0.472453
C	1.984503	-0.116554	-1.384717
O	2.357876	1.061115	-0.783790
H	3.059079	-1.970185	-1.896552
H	5.200535	-0.625933	-0.756804
H	0.935030	-0.199710	-1.653475
H	4.135641	1.818751	0.009840
C	-0.255454	-0.471954	2.924146
C	1.154030	-0.195317	2.871746
C	1.624528	-0.798350	1.719628
C	-0.542883	-1.223142	1.800975
O	0.597706	-1.433168	1.066607
H	-0.973015	-0.145766	3.673995
H	1.749313	0.376461	3.580984
H	-1.452906	-1.650933	1.388548
H	2.601241	-0.866280	1.247109
C	-2.584184	-2.766531	-0.743456
C	-3.281128	-1.512056	-0.643328
C	-2.392262	-0.539419	-1.057137
C	-1.320627	-2.465644	-1.213601
O	-1.197167	-1.114869	-1.412886
H	-2.956424	-3.760243	-0.501966
H	-4.302007	-1.341286	-0.307392
H	-0.438998	-3.060939	-1.434506
H	-2.447028	0.541254	-1.158086
C	-1.835550	3.040013	-0.965885

C	-0.532069	2.443194	-0.869038
C	-0.504496	1.760883	0.332827
C	-2.506168	2.682530	0.189809
O	-1.703832	1.908434	0.986229
H	-2.233330	3.654092	-1.771640
H	0.288174	2.507487	-1.579911
H	-3.499197	2.894790	0.577389
H	0.252288	1.180051	0.851701

Cartesian coordinates of : Furan4_15

Atomic number (AN) and Cartesian coordinates

AN	X	Y	Z
O	-1.130617	-1.204780	-1.808992
C	-1.053684	-1.596159	-0.493369
H	-0.070404	-1.525548	-0.039403
C	-2.296908	-1.997072	-0.046006
H	-2.524102	-2.351092	0.956599
C	-3.198685	-1.836036	-1.153855
H	-4.265622	-2.048625	-1.186102
C	-2.437497	-1.349045	-2.199824
H	-2.659678	-1.079083	-3.228833
O	1.428369	-1.311951	1.880637
C	1.115781	0.007841	1.645261
H	1.844144	0.565990	1.062506
C	-0.099615	0.321159	2.220147
H	-0.595676	1.287756	2.170244
C	-0.569089	-0.882054	2.848003
H	-1.494581	-1.028224	3.401957
C	0.393765	-1.844014	2.610054
H	0.494034	-2.892143	2.879679
O	2.158765	-1.016777	-1.144753
C	1.838976	0.192194	-1.709956
H	0.873886	0.238147	-2.204639
C	2.855378	1.102179	-1.491104
H	2.877411	2.132013	-1.842935
C	3.863483	0.407409	-0.735463
H	4.814744	0.793756	-0.374987
C	3.386931	-0.876155	-0.551921
H	3.771950	-1.755464	-0.043488
O	-0.713539	1.623704	-0.650170
C	-0.171119	2.750791	-0.091358
H	0.877888	2.928375	-0.312068
C	-1.120683	3.418977	0.658766
H	-0.965734	4.344559	1.209868
C	-2.326153	2.641822	0.556827
H	-3.292097	2.846580	1.014482
C	-2.021489	1.558227	-0.245115
H	-2.586337	0.701046	-0.603162

Cartesian coordinates of : Furan4_16

Atomic number (AN) and Cartesian coordinates

AN	X	Y	Z
C	1.029547	-0.522352	3.052231
C	1.492767	0.227022	1.917392
C	0.415528	0.975165	1.482510
C	-0.296568	-0.172019	3.228646
O	-0.677644	0.743258	2.281789
H	1.589739	-1.228376	3.662317
H	2.480193	0.202908	1.460241
H	-1.060193	-0.460015	3.946493
H	0.265699	1.675829	0.665608
C	-0.777708	2.007544	-1.859212
C	-0.996696	3.168574	-1.041275
C	-1.942678	2.801667	-0.102166
C	-1.601782	1.018525	-1.354502
O	-2.317066	1.496981	-0.286763
H	-0.097083	1.907970	-2.702438
H	-0.523720	4.144805	-1.126215
H	-1.809273	-0.011877	-1.633467
H	-2.421080	3.319812	0.724411
C	3.909221	-0.782867	-0.703925
C	2.651052	-1.395562	-1.031380
C	1.771734	-0.360592	-1.277178
C	3.702915	0.581980	-0.777901
O	2.403803	0.849206	-1.130085
H	4.846940	-1.273133	-0.448914
H	2.409608	-2.455531	-1.074413
H	4.342998	1.447734	-0.629490
H	0.718953	-0.315851	-1.530309
C	-2.916454	-2.220138	-0.685157
C	-2.283213	-1.734062	0.509196
C	-0.922712	-1.820580	0.291895
C	-1.894678	-2.570669	-1.547740
O	-0.675621	-2.334808	-0.960894
H	-3.981855	-2.299451	-0.892704
H	-2.757926	-1.343012	1.405926
H	-1.866606	-2.992375	-2.549051
H	-0.045800	-1.566342	0.881014

Cartesian coordinates of : Furan4_17

Atomic number (AN) and Cartesian coordinates

AN	X	Y	Z
C	-0.606863	-0.908049	2.954795
C	-0.306290	0.260608	2.176222
C	0.960191	0.067215	1.661143
C	0.499921	-1.730902	2.861016

O	1.463299	-1.144994	2.077636
H	-1.514309	-1.122929	3.516506
H	-0.943785	1.124443	1.998164
H	0.752950	-2.702446	3.277652
H	1.613835	0.657408	1.024657
C	-2.149737	-1.949853	-0.120503
C	-2.749150	-1.814773	-1.419372
C	-1.705863	-1.783535	-2.324724
C	-0.785994	-1.989744	-0.324989
O	-0.505140	-1.895827	-1.668287
H	-2.645072	-1.993241	0.846619
H	-3.807476	-1.750238	-1.665777
H	0.071685	-2.062168	0.336823
H	-1.650058	-1.702383	-3.406959
C	3.584686	-0.384244	-0.621391
C	3.608498	1.053046	-0.617768
C	2.438094	1.459056	-1.230064
C	2.402251	-0.748241	-1.234441
O	1.697855	0.368616	-1.611407
H	4.330378	-1.065750	-0.217583
H	4.380129	1.707498	-0.217262
H	1.940509	-1.699413	-1.480686
H	1.999176	2.427121	-1.451860
C	-2.568976	1.572862	-0.314165
C	-2.500837	2.773116	0.473246
C	-1.179474	3.177044	0.442763
C	-1.287369	1.329217	-0.765239
O	-0.434129	2.307999	-0.313320
H	-3.439522	0.954791	-0.519811
H	-3.310507	3.278522	0.996311
H	-0.831613	0.549949	-1.368234
H	-0.639939	4.015551	0.875283

Cartesian coordinates of : Furan4_18

Atomic number (AN) and Cartesian coordinates

AN	X	Y	Z
C	-1.551035	2.149777	1.230153
C	-2.306998	3.002064	0.354328
C	-1.666939	2.957511	-0.870413
C	-0.507046	1.646112	0.479792
O	-0.567716	2.139305	-0.802173
H	-1.748906	1.918328	2.274074
H	-3.203345	3.574753	0.585195
H	0.319458	0.973280	0.689323
H	-1.849072	3.430869	-1.831730
C	1.892299	0.195787	-1.522129
C	2.576468	1.304388	-0.915728
C	3.797735	0.820739	-0.489821
C	2.749223	-0.881403	-1.423172
O	3.916634	-0.513899	-0.796025

H	0.890777	0.185853	-1.945290
H	2.214552	2.324331	-0.804614
H	2.688100	-1.923404	-1.725028
H	4.653383	1.268486	0.008597
C	1.097383	-0.390949	2.884102
C	-0.297680	-0.735140	2.909126
C	-0.547898	-1.420794	1.735622
C	1.597502	-0.892673	1.696867
O	0.602769	-1.528541	0.996426
H	1.664327	0.158304	3.633244
H	-1.027751	-0.507880	3.683636
H	2.579278	-0.888259	1.231928
H	-1.435914	-1.861549	1.290100
C	-3.317147	-1.573082	-0.676141
C	-2.505259	-2.716631	-0.997639
C	-1.285131	-2.215197	-1.407919
C	-2.531572	-0.461621	-0.912723
O	-1.294214	-0.845509	-1.364960
H	-4.344167	-1.564447	-0.316229
H	-2.776842	-3.768696	-0.936402
H	-2.692188	0.609206	-0.816702
H	-0.354916	-2.673362	-1.732084

Cartesian coordinates of : Furan4_19

Atomic number (AN) and Cartesian coordinates

AN	X	Y	Z
C	-1.079289	-1.309354	-1.695505
C	-0.360405	-2.334770	-2.399902
C	-0.208210	-3.381191	-1.508440
C	-1.310003	-1.802061	-0.426431
O	-0.786866	-3.067485	-0.305277
H	-1.379995	-0.330684	-2.060648
H	-0.002920	-2.317441	-3.427950
H	-1.803508	-1.409641	0.458266
H	0.250984	-4.363915	-1.573630
C	2.334947	-0.360289	2.399884
C	1.309469	-1.079113	1.695514
C	1.802144	-1.309892	0.426441
C	3.381368	-0.208193	1.508403
O	3.067602	-0.786850	0.305257
H	2.317665	-0.002791	3.427928
H	0.330764	-1.379703	2.060653
H	4.364144	0.250889	1.573592
H	1.409658	-1.803362	-0.458244
C	1.079230	1.309282	-1.695452
C	0.360371	2.334644	-2.399954
C	0.208159	3.381141	-1.508583
C	1.309920	1.802096	-0.426416
O	0.786788	3.067534	-0.305382
H	1.379935	0.330579	-2.060504

H	0.002901	2.317225	-3.428006
H	1.803378	1.409744	0.458336
H	-0.250997	4.363875	-1.573885
C	-2.334735	0.360335	2.399950
C	-1.309409	1.079280	1.695485
C	-1.802225	1.309993	0.426454
C	-3.381216	0.208101	1.508565
O	-3.067631	0.786794	0.305391
H	-2.317306	0.002829	3.427989
H	-0.330732	1.380031	2.060570
H	-4.363918	-0.251130	1.573832
H	-1.409902	1.803504	-0.458282

Cartesian coordinates of : Furan4_20

Atomic number (AN) and Cartesian coordinates

AN	X	Y	Z
C	-1.613778	-2.649222	-0.358813
C	-0.545142	-3.533260	-0.738122
C	0.268982	-2.809314	-1.587223
C	-1.371498	-1.451277	-1.002008
O	-0.228104	-1.542092	-1.757654
H	-2.450502	-2.853070	0.305444
H	-0.386406	-4.563639	-0.426590
H	-1.881240	-0.492895	-1.020402
H	1.192751	-3.031804	-2.113518
C	1.648752	2.535608	-0.404223
C	1.370015	1.343293	-1.155088
C	0.202455	1.592923	-1.848633
C	0.630177	3.423602	-0.696785
O	-0.255200	2.859623	-1.579606
H	2.485304	2.715864	0.267212
H	1.940035	0.417884	-1.172924
H	0.402630	4.438502	-0.381851
H	-0.394438	1.010981	-2.544471
C	-1.780224	0.190504	1.964449
C	-3.161021	-0.103749	1.699850
C	-3.526398	0.699260	0.636622
C	-1.400647	1.147505	1.042991
O	-2.463642	1.468062	0.232833
H	-1.145840	-0.255839	2.726973
H	-3.807756	-0.806830	2.221423
H	-0.469380	1.666798	0.830655
H	-4.454134	0.847039	0.089997
C	1.765730	-0.071736	1.933995
C	3.165300	0.140995	1.691711
C	3.520124	-0.747223	0.695231
C	1.366228	-1.072011	1.068861
O	2.434384	-1.495127	0.314239
H	1.133260	0.452636	2.646671
H	3.830363	0.848419	2.183621

H	0.417789	-1.562447	0.866694
H	4.453731	-0.970717	0.185689

Cartesian coordinates of : Furan4_21

Atomic number (AN) and Cartesian coordinates

AN	X	Y	Z
C	-3.629541	1.011357	-0.988245
C	-3.810276	-0.345014	-0.548330
C	-2.594539	-0.974882	-0.740183
C	-2.318506	1.104355	-1.417214
O	-1.684865	-0.101285	-1.274367
H	-4.362898	1.815462	-0.993222
H	-4.710582	-0.801314	-0.141272
H	-1.717297	1.904071	-1.841295
H	-2.225516	-1.978977	-0.549799
C	-0.790648	1.804147	2.570088
C	-1.398097	0.701272	1.877649
C	-0.426102	-0.277271	1.784245
C	0.506440	1.414309	2.848734
O	0.734993	0.147323	2.379900
H	-1.241130	2.759773	2.831693
H	-2.407016	0.634762	1.476986
H	1.339575	1.888882	3.360552
H	-0.398470	-1.272480	1.348125
C	3.120301	1.757176	-1.321090
C	2.337188	2.023741	-0.145641
C	1.032804	1.700991	-0.465035
C	2.233093	1.295029	-2.274830
O	0.960276	1.261128	-1.764972
H	4.192771	1.884386	-1.454864
H	2.681625	2.394229	0.817225
H	2.340757	0.974372	-3.307709
H	0.100648	1.698046	0.092512
C	0.260281	-3.228647	-0.491785
C	0.746070	-2.049208	-1.153979
C	1.732191	-1.529270	-0.338948
C	0.988220	-3.340540	0.679488
O	1.890739	-2.314096	0.776691
H	-0.509927	-3.919829	-0.830533
H	0.403803	-1.615824	-2.090163
H	0.996904	-4.060424	1.493428
H	2.374345	-0.654975	-0.399225

Cartesian coordinates of : Furan4_22

Atomic number (AN) and Cartesian coordinates

AN	X	Y	Z

```
  C     -0.004815     -3.122203      1.299225
  C      1.172738     -3.065235      0.475525
  C      1.736524     -1.822513      0.692489
  C     -0.073579     -1.909076      1.954944
  O      0.985962     -1.113614      1.596440
  H     -0.720800     -3.936567      1.387478
  H      1.556478     -3.831778     -0.194709
  H     -0.784051     -1.466350      2.646746
  H      2.616043     -1.316833      0.302393
  C     -1.172815      3.065002      0.475658
  C      0.004719      3.121914      1.299390
  C      0.073326      1.908840      1.955222
  C     -1.736747      1.822365      0.692720
  O     -0.986293      1.113463      1.596759
  H     -1.556445      3.831523     -0.194663
  H      0.720799      3.936200      1.387579
  H     -2.616320      1.316754      0.302654
  H      0.783731      1.466101      2.647085
  C      3.231845      0.051739     -1.987452
  C      1.805445      0.187903     -2.079615
  C      1.409237      0.920465     -0.976296
  C      3.603797      0.713433     -0.832071
  O      2.502671      1.247446     -0.212917
  H      3.901692     -0.462112     -2.674432
  H      1.154019     -0.213357     -2.852396
  H      4.558489      0.897629     -0.346188
  H      0.450778      1.283051     -0.615958
  C     -3.231454     -0.051316     -1.987787
  C     -1.805044     -0.187535     -2.079685
  C     -1.409105     -0.920367     -0.976451
  C     -3.603680     -0.713247     -0.832630
  O     -2.502712     -1.247456     -0.213369
  H     -3.901131      0.462738     -2.674782
  H     -1.153431      0.213861     -2.852236
  H     -4.558482     -0.897498     -0.346985
  H     -0.450738     -1.283087     -0.616005
 ---------------------------------------------------
```

Cartesian coordinates of : Furan4_23

```
 ---------------------------------------------------
 Atomic number (AN) and Cartesian coordinates
  AN          X            Y             Z
 ---------------------------------------------------
  C     -0.251778     -3.548228      1.199212
  C     -0.979285     -3.179967      0.014691
  C     -1.388275     -1.874059      0.199651
  C     -0.272589     -2.436386      2.020105
  O     -0.965934     -1.415966      1.423210
  H      0.225893     -4.500100      1.423430
  H     -1.170313     -3.789384     -0.866170
  H      0.137600     -2.221701      3.003448
  H     -1.953787     -1.166738     -0.399973
  C      1.428163      2.904060      1.165828
```

C	0.332795	2.780724	2.088727
C	0.171170	1.426203	2.311281
C	1.854703	1.616370	0.897732
O	1.096988	0.712204	1.597539
H	1.847860	3.816170	0.746333
H	-0.266538	3.577563	2.524892
H	2.631567	1.190736	0.267868
H	-0.525233	0.838188	2.902038
C	-3.511192	0.900483	-0.894290
C	-2.550107	1.416678	0.041767
C	-1.341254	1.458824	-0.624813
C	-2.817601	0.669655	-2.067292
O	-1.498047	1.010997	-1.914317
H	-4.571570	0.716586	-0.732896
H	-2.714301	1.710326	1.075675
H	-3.101769	0.294150	-3.046695
H	-0.336168	1.762136	-0.347714
C	3.010758	0.030801	-2.136923
C	3.171927	-0.987141	-1.133418
C	1.903252	-1.268804	-0.661156
C	1.654740	0.293994	-2.200076
O	0.976446	-0.496169	-1.310049
H	3.783771	0.506952	-2.737320
H	4.096451	-1.455209	-0.800601
H	1.040669	0.963149	-2.796610
H	1.507674	-1.950585	0.087086

Cartesian coordinates of : Furan4_24

Atomic number (AN) and Cartesian coordinates

AN	X	Y	Z
C	4.060776	0.612696	-0.582656
C	3.877630	-0.765608	-0.950706
C	2.549232	-0.895833	-1.305292
C	2.830250	1.219437	-0.740562
O	1.905008	0.308985	-1.187177
H	4.973795	1.098231	-0.243643
H	4.620667	-1.560639	-0.953596
H	2.462470	2.230777	-0.588294
H	1.935168	-1.732031	-1.627455
C	-1.197426	-1.134798	-1.492889
C	-1.329659	-2.558782	-1.365235
C	-2.532131	-2.778490	-0.719624
C	-2.327393	-0.588422	-0.917179
O	-3.150647	-1.583592	-0.446644
H	-0.373473	-0.569379	-1.919779
H	-0.635898	-3.330750	-1.693159
H	-2.680410	0.429028	-0.771747
H	-3.067632	-3.672159	-0.410083
C	-1.862783	2.990169	-1.091721
C	-0.609345	2.289271	-1.057359

C	-0.494424	1.758429	0.213906
C	-2.419602	2.838941	0.164785
O	-1.590583	2.097630	0.966828
H	-2.305363	3.530835	-1.926180
H	0.105609	2.165218	-1.867580
H	-3.339817	3.188270	0.625458
H	0.251814	1.150979	0.716979
C	-0.381280	-0.439703	2.864362
C	0.994554	-0.024788	2.897158
C	1.608172	-0.637089	1.819902
C	-0.507341	-1.271346	1.768860
O	0.701012	-1.403574	1.131660
H	-1.181986	-0.151432	3.542004
H	1.474681	0.641265	3.611458
H	-1.333713	-1.823405	1.329705
H	2.620393	-0.634895	1.424079

Cartesian coordinates of : Furan4_25

Atomic number (AN) and Cartesian coordinates

AN	X	Y	Z
C	-0.618201	-0.935170	2.833796
C	-0.053750	0.246202	2.243563
C	1.123518	-0.148953	1.639571
C	0.254417	-1.966920	2.545029
O	1.322382	-1.498747	1.820094
H	-1.545370	-1.021530	3.397423
H	-0.465963	1.252679	2.234102
H	0.267663	-3.028947	2.775643
H	1.888125	0.369820	1.066773
C	2.799335	0.987624	-1.507165
C	1.832452	-0.025004	-1.829950
C	2.273310	-1.180153	-1.215305
C	3.754967	0.374590	-0.717777
O	3.444051	-0.948708	-0.536791
H	2.808758	2.028045	-1.827100
H	0.919867	0.078412	-2.410860
H	4.674455	0.713893	-0.248210
H	1.891713	-2.195889	-1.161441
C	-0.832823	3.441860	0.762481
C	-2.098827	2.779058	0.604531
C	-1.871985	1.707599	-0.238466
C	0.070922	2.723776	0.001890
O	-0.554264	1.672520	-0.615278
H	-0.609746	4.326516	1.355871
H	-3.053787	3.049903	1.050787
H	1.136005	2.817130	-0.189327
H	-2.505056	0.923786	-0.646799
C	-3.385459	-1.617573	-1.154783
C	-2.492153	-1.792778	-0.042715
C	-1.233766	-1.450397	-0.496476

C	-2.604540	-1.183674	-2.209552
O	-1.292983	-1.083405	-1.819872
H	-4.460318	-1.786246	-1.183897
H	-2.734364	-2.113498	0.967475
H	-2.814445	-0.929474	-3.245140
H	-0.255540	-1.378408	-0.032548

Cartesian coordinates of : Furan4_26

Atomic number (AN) and Cartesian coordinates

AN	X	Y	Z
C	-1.185104	1.310322	2.001467
C	-2.598599	1.396709	2.242388
C	-3.174600	1.710525	1.025424
C	-1.001155	1.572253	0.656549
O	-2.210191	1.825225	0.057278
H	-0.405978	1.074539	2.723041
H	-3.128052	1.246929	3.181226
H	-0.131281	1.611423	0.006671
H	-4.196745	1.888826	0.702142
C	-1.051736	-3.341349	-0.846739
C	-2.071089	-2.331635	-0.932733
C	-1.948680	-1.557526	0.203617
C	-0.382770	-3.110173	0.340648
O	-0.926237	-2.031233	0.990402
H	-0.833913	-4.134495	-1.559387
H	-2.789588	-2.175546	-1.734344
H	0.438821	-3.606908	0.850263
H	-2.478240	-0.691021	0.589966
C	0.948360	1.919157	-2.368868
C	1.499574	0.724636	-1.791838
C	0.469812	-0.196288	-1.752533
C	-0.377382	1.636712	-2.643384
O	-0.675603	0.350350	-2.277799
H	1.452003	2.865420	-2.556829
H	2.512708	0.558178	-1.432261
H	-1.196285	2.211538	-3.067023
H	0.388710	-1.222644	-1.404723
C	2.923525	-1.167346	0.817368
C	1.876939	-0.385087	1.413002
C	2.342829	0.914592	1.442665
C	3.950460	-0.288036	0.530695
O	3.606384	0.985097	0.910212
H	2.928406	-2.237463	0.618582
H	0.898238	-0.726412	1.739207
H	4.939188	-0.401350	0.094060
H	1.924887	1.858681	1.782285

Cartesian coordinates of : Furan4_27

```
 ---------------------------------------------------
 Atomic number (AN) and Cartesian coordinates
  AN         X            Y            Z
 ---------------------------------------------------
  C     -2.028999     1.923586    -0.464398
  C     -3.396492     1.767950    -0.052980
  C     -3.762375     0.486708    -0.418701
  C     -1.658118     0.725896    -1.046979
  O     -2.713534    -0.152105    -1.029364
  H     -1.390512     2.793046    -0.327093
  H     -4.030969     2.495488     0.449307
  H     -0.735393     0.335142    -1.466889
  H     -4.684049    -0.083265    -0.336425
  C     -0.789766    -1.313233     2.931349
  C     -1.087260    -0.262890     1.998714
  C      0.111281     0.373780     1.738755
  C      0.569087    -1.238223     3.177075
  O      1.126297    -0.209915     2.459220
  H     -1.477585    -2.033581     3.370942
  H     -2.052985    -0.002246     1.570056
  H      1.250239    -1.798331     3.812602
  H      0.398651     1.213248     1.113086
  C      1.983724     2.958672    -0.230120
  C      2.869319     2.000284     0.373080
  C      2.661964     0.808822    -0.292821
  C      1.304986     2.279479    -1.224278
  O      1.710945     0.970278    -1.271035
  H      1.863147     4.010024     0.023716
  H      3.559342     2.156453     1.199474
  H      0.558179     2.569348    -1.958835
  H      3.071634    -0.191796    -0.199011
  C     -0.475478    -2.360671    -0.813673
  C     -0.191976    -2.258958    -2.219574
  C      1.174688    -2.089455    -2.324987
  C      0.736344    -2.242611    -0.166213
  O      1.751856    -2.086891    -1.080207
  H     -1.444637    -2.477467    -0.335594
  H     -0.897813    -2.302172    -3.046896
  H      1.028292    -2.245677     0.880492
  H      1.854361    -1.963971    -3.163347
 ---------------------------------------------------
```

Cartesian coordinates of : Furan4_28

```
 ---------------------------------------------------
 Atomic number (AN) and Cartesian coordinates
  AN         X            Y            Z
 ---------------------------------------------------
  C     -3.193810     0.838750     1.629184
  C     -3.436277    -0.149551     0.614304
  C     -2.366936    -1.024346     0.660148
  C     -1.993280     0.491183     2.219138
  O     -1.485498    -0.642535     1.640582
  H     -3.812402     1.694864     1.891025
```

H	-4.281870	-0.211504	-0.067641
H	-1.409379	0.912719	3.033255
H	-2.092789	-1.914048	0.099206
C	1.753745	0.353843	1.631114
C	2.521241	-0.713619	2.211915
C	3.404964	-1.126373	1.232052
C	2.223236	0.513325	0.342892
O	3.232331	-0.384149	0.092018
H	0.949260	0.926915	2.083079
H	2.439410	-1.130277	3.213969
H	1.963636	1.179501	-0.474070
H	4.179497	-1.887485	1.190832
C	0.704463	3.558883	-0.837104
C	-0.228431	2.907428	0.041161
C	-0.719159	1.817376	-0.650666
C	0.709820	2.820222	-2.004955
O	-0.156537	1.760957	-1.902221
H	1.299329	4.448915	-0.641698
H	-0.508556	3.197187	1.051641
H	1.236134	2.914329	-2.951040
H	-1.431921	1.032910	-0.417184
C	-0.155690	-3.318579	-0.943142
C	-0.887217	-2.873009	-2.098685
C	-0.656431	-1.512928	-2.195909
C	0.464900	-2.198323	-0.421615
O	0.166643	-1.098573	-1.182880
H	-0.091387	-4.328967	-0.543746
H	-1.499067	-3.468500	-2.773773
H	1.106093	-2.018796	0.437580
H	-0.972322	-0.739044	-2.890425

Cartesian coordinates of : Furan4_29

Atomic number (AN) and Cartesian coordinates

AN	X	Y	Z
C	2.198486	0.675919	-1.755227
C	2.363115	-0.742385	-1.594467
C	3.303638	-0.910230	-0.598185
C	3.054259	1.265941	-0.844146
O	3.733352	0.308181	-0.134724
H	1.529550	1.200876	-2.433727
H	1.843118	-1.541149	-2.117660
H	3.305498	2.295449	-0.603510
H	3.736407	-1.780309	-0.113150
C	-2.887070	-1.948614	-0.923360
C	-2.660256	-1.497498	-2.269823
C	-1.333722	-1.110723	-2.322936
C	-1.685292	-1.800955	-0.256780
O	-0.734013	-1.296088	-1.105946
H	-3.812366	-2.328938	-0.494959
H	-3.372093	-1.458932	-3.092104

H	-1.362282	-1.987508	0.764256
H	-0.701776	-0.702280	-3.107233
C	-1.273119	3.335201	0.832086
C	-2.425981	2.530539	0.530848
C	-1.998936	1.556358	-0.350583
C	-0.228657	2.790684	0.109280
O	-0.662199	1.712660	-0.617784
H	-1.212725	4.198120	1.492520
H	-3.438208	2.645776	0.913601
H	0.826126	3.032121	0.013108
H	-2.486801	0.724610	-0.851898
C	-0.582556	-0.980718	3.159938
C	-0.293676	0.123082	2.286516
C	0.681217	-0.318358	1.414620
C	0.237954	-2.017421	2.754767
O	1.012845	-1.623600	1.692404
H	-1.297702	-1.016885	3.979849
H	-0.752449	1.109430	2.272946
H	0.394497	-3.033880	3.106393
H	1.184916	0.129355	0.564687

Cartesian coordinates of : Furan4_30

Atomic number (AN) and Cartesian coordinates

AN	X	Y	Z
C	-3.154844	1.046964	-0.067902
C	-2.859332	0.479838	-1.354120
C	-3.713045	-0.593482	-1.507390
C	-4.164229	0.274305	0.470753
O	-4.514855	-0.731170	-0.398514
H	-2.682638	1.905827	0.404484
H	-2.097947	0.800203	-2.060802
H	-4.718660	0.304495	1.404813
H	-3.868601	-1.324747	-2.295928
C	3.228524	0.487276	-1.353527
C	3.927108	-0.750331	-1.567803
C	4.133614	-1.294789	-0.314288
C	3.058680	0.605766	0.011832
O	3.612181	-0.474609	0.653082
H	2.884705	1.197982	-2.101817
H	4.238994	-1.188584	-2.513753
H	2.582721	1.345840	0.649208
H	4.615075	-2.202286	0.040159
C	-0.644663	-2.335489	1.639617
C	0.741874	-2.232560	1.275958
C	0.764111	-1.798136	-0.035486
C	-1.367868	-1.954666	0.523692
O	-0.515755	-1.633381	-0.502892
H	-1.064302	-2.642897	2.595560
H	1.614636	-2.428051	1.895329
H	-2.428492	-1.867262	0.306681

```
  H      1.559583     -1.563924     -0.737591
  C      0.723837      3.291089      0.777488
  C      0.328066      2.267162      1.706553
  C     -0.074981      1.185743      0.946360
  C      0.528568      2.756070     -0.482346
  O      0.040727      1.478702     -0.388566
  H      1.099972      4.288037      0.998653
  H      0.340294      2.312743      2.793744
  H      0.675867      3.137278     -1.489516
  H     -0.462107      0.200457      1.189552
 ---------------------------------------------------

Cartesian coordinates of :  Furan4_31

 ---------------------------------------------------
 Atomic number (AN) and Cartesian coordinates
  AN         X             Y             Z
 ---------------------------------------------------
  C      4.198513     -0.755936      1.106800
  C      3.538082      0.489868      1.388707
  C      3.084079      0.970220      0.175986
  C      4.096134     -0.940867     -0.258768
  O      3.422663      0.105152     -0.833633
  H      4.688317     -1.428298      1.808589
  H      3.411372      0.974503      2.354667
  H      4.442166     -1.714803     -0.938615
  H      2.539322      1.861222     -0.125318
  C      0.937564     -2.454104     -0.868565
  C     -0.423713     -2.563393     -1.316929
  C     -1.207749     -2.039714     -0.305022
  C      0.884779     -1.873824      0.385316
  O     -0.417705     -1.626978      0.737406
  H      1.841534     -2.746709     -1.398007
  H     -0.786436     -2.964999     -2.261038
  H      1.643353     -1.561891      1.097828
  H     -2.277383     -1.898356     -0.171332
  C     -4.042281     -0.613484      1.304467
  C     -3.009081      0.384156      1.279808
  C     -2.989219      0.887813     -0.005555
  C     -4.575738     -0.646473      0.029530
  O     -3.942949      0.267141     -0.774832
  H     -4.354141     -1.232688      2.143400
  H     -2.342136      0.675914      2.087393
  H     -5.370373     -1.221227     -0.438617
  H     -2.392687      1.642898     -0.510501
  C      0.119692      3.260612     -0.623737
  C      0.174444      2.199687     -1.592075
  C      0.122336      1.017943     -0.877427
  C      0.037826      2.643226      0.611888
  O      0.037475      1.281574      0.465180
  H      0.135660      4.333896     -0.803071
  H      0.244939      2.287906     -2.674334
  H     -0.029096      3.012662      1.631939
  H      0.135707     -0.029510     -1.162507
```

Cartesian coordinates of : Furan4_32

AN	X	Y	Z
C	-0.269419	1.720148	0.864777
C	-0.987338	2.947159	0.657244
C	-1.022275	3.146022	-0.710421
C	0.081020	1.258245	-0.388688
O	-0.370542	2.124474	-1.355329
H	-0.055049	1.216462	1.804947
H	-1.422863	3.604544	1.407608
H	0.618690	0.385034	-0.748219
H	-1.431717	3.925746	-1.347032
C	4.239064	0.210026	1.274299
C	4.823784	-0.709157	0.336247
C	4.215020	-0.454377	-0.877994
C	3.317879	0.953942	0.562157
O	3.302137	0.559433	-0.750890
H	4.462244	0.314086	2.334244
H	5.590113	-1.458809	0.523336
H	2.626463	1.750941	0.821191
H	4.323581	-0.873457	-1.874684
C	-0.440310	-2.201853	-0.904216
C	0.996788	-2.211356	-0.920477
C	1.402054	-1.783593	0.329982
C	-0.808806	-1.768051	0.354147
O	0.306782	-1.516910	1.114751
H	-1.123009	-2.459628	-1.710971
H	1.654900	-2.491921	-1.740352
H	-1.764744	-1.589302	0.838908
H	2.372102	-1.621197	0.791488
C	-4.293368	-0.823838	1.113030
C	-3.607735	0.421756	0.903494
C	-3.150310	0.397126	-0.398826
C	-4.204819	-1.515845	-0.080334
O	-3.515128	-0.777754	-1.008203
H	-4.787752	-1.172604	2.017575
H	-3.454865	1.231357	1.613419
H	-4.566293	-2.484141	-0.416041
H	-2.575165	1.090825	-1.004573

Atomic number (AN) and Cartesian coordinates

Cartesian coordinates of : Furan4_33

Atomic number (AN) and Cartesian coordinates

AN	X	Y	Z
C	-3.648492	-0.478081	-1.346595
C	-4.352664	0.694269	-0.903230

C	-4.114162	0.791151	0.454201
C	-3.034512	-1.007112	-0.228353
O	-3.316175	-0.240839	0.874505
H	-3.598251	-0.885179	-2.354479
H	-4.957456	1.377058	-1.496998
H	-2.407207	-1.877147	-0.053299
H	-4.434674	1.488509	1.223708
C	0.035246	-3.235376	0.461008
C	-0.096872	-2.233498	1.483231
C	-0.069046	-1.012717	0.835607
C	0.131788	-2.546908	-0.736222
O	0.069192	-1.197615	-0.514768
H	0.057589	-4.316822	0.580770
H	-0.202910	-2.384703	2.555522
H	0.251492	-2.853998	-1.771814
H	-0.151177	0.015544	1.170517
C	3.974147	-0.187409	0.889030
C	4.522028	0.822208	0.024216
C	3.913006	0.644104	-1.203337
C	3.073544	-0.904014	0.125742
O	3.032677	-0.405715	-1.152104
H	4.207998	-0.367242	1.936437
H	5.265502	1.579448	0.265819
H	2.426758	-1.754196	0.325320
H	3.996960	1.147828	-2.162732
C	0.397339	1.773403	-0.891474
C	-0.981084	1.942947	-0.526439
C	-0.981372	2.322088	0.802119
C	1.138359	2.051502	0.239862
O	0.305766	2.393499	1.280981
H	0.799426	1.448559	-1.848148
H	-1.866156	1.774888	-1.135816
H	2.201103	2.028989	0.464671
H	-1.764192	2.564478	1.515415

Cartesian coordinates of : Furan4_34

Atomic number (AN) and Cartesian coordinates

AN	X	Y	Z
C	-3.717958	-0.109175	0.751847
C	-4.190265	-0.990614	-0.280459
C	-3.791328	-0.426178	-1.477145
C	-3.067486	0.925576	0.109899
O	-3.108351	0.741789	-1.249382
H	-3.835711	-0.216687	1.828214
H	-4.750471	-1.916945	-0.166872
H	-2.548322	1.817374	0.451410
H	-3.919331	-0.706236	-2.519464
C	-0.930779	-2.461045	0.963006
C	0.396369	-2.628415	1.490015
C	1.258888	-2.136635	0.526389

C	-0.779467	-1.881065	-0.282469
O	0.549604	-1.690316	-0.558230
H	-1.877775	-2.715736	1.434084
H	0.686074	-3.047085	2.451863
H	-1.481195	-1.542497	-1.039330
H	2.337784	-2.023135	0.458357
C	-0.040918	2.190187	1.724761
C	-0.103994	3.180368	0.684098
C	-0.137078	2.478017	-0.508515
C	-0.039720	0.961643	1.090001
O	-0.098514	1.132326	-0.267259
H	0.002351	2.354757	2.799653
H	-0.121415	4.263410	0.789345
H	-0.000216	-0.065683	1.440625
H	-0.186272	2.771496	-1.553488
C	4.021633	0.330137	0.581637
C	4.440948	-0.668492	-0.364435
C	3.620965	-0.516997	-1.467051
C	2.980174	1.014025	-0.015518
O	2.733778	0.506162	-1.264638
H	4.429009	0.524538	1.571772
H	5.238530	-1.401142	-0.255625
H	2.351687	1.846325	0.290643
H	3.543230	-1.030460	-2.421479

Cartesian coordinates of : Furan4_35

Atomic number (AN) and Cartesian coordinates

AN	X	Y	Z
C	4.430058	0.370356	0.403382
C	4.749333	-0.658153	-0.549017
C	3.770531	-0.595700	-1.522784
C	3.280801	0.981449	-0.059252
O	2.875604	0.401641	-1.234764
H	4.969200	0.628840	1.312505
H	5.585242	-1.354536	-0.526446
H	2.654185	1.790446	0.305701
H	3.579245	-1.156299	-2.433705
C	-0.600940	-2.118299	1.494157
C	0.791665	-2.129301	1.849037
C	1.485398	-1.771431	0.707726
C	-0.656269	-1.756038	0.162700
O	0.609932	-1.551791	-0.326532
H	-1.457132	-2.333534	2.129995
H	1.233944	-2.364217	2.815360
H	-1.469980	-1.617341	-0.542823
H	2.539142	-1.644925	0.473126
C	-3.906619	-0.618812	0.536896
C	-4.176511	-1.552195	-0.521771
C	-3.616305	-1.008610	-1.662354
C	-3.199938	0.421812	-0.033169

```
  O     -3.022448      0.194843     -1.375165
  H     -4.188194     -0.698731      1.584938
  H     -4.707789     -2.500010     -0.460524
  H     -2.771211      1.340494      0.354191
  H     -3.566033     -1.327175     -2.700112
  C     -0.064445      1.624646     -0.444157
  C      0.002622      1.319782      0.958907
  C     -0.701536      2.317666      1.602463
  C     -0.803815      2.785216     -0.551828
  O     -1.201019      3.219832      0.691376
  H      0.368873      1.055236     -1.263095
  H      0.488409      0.467727      1.431397
  H     -1.127722      3.396938     -1.389431
  H     -0.930897      2.526546      2.643987
 ---------------------------------------------------
```

Cartesian coordinates of : Furan4_36

```
 ---------------------------------------------------
 Atomic number (AN) and Cartesian coordinates
  AN         X            Y            Z
 ---------------------------------------------------
  C     -3.403507      0.291576      1.260948
  C     -4.193218     -0.909073      1.279074
  C     -4.287545     -1.325901     -0.035320
  C     -3.073155      0.518040     -0.060060
  O     -3.613466     -0.459734     -0.858412
  H     -3.101869      0.906928      2.105644
  H     -4.635376     -1.407029      2.139835
  H     -2.500570      1.287878     -0.569068
  H     -4.775991     -2.164175     -0.524887
  C      3.341469      0.866195     -0.373176
  C      3.284723      0.789127      1.060198
  C      4.005967     -0.333069      1.415393
  C      4.094052     -0.214442     -0.785533
  O      4.507353     -0.953568      0.296662
  H      2.875658      1.608632     -1.017950
  H      2.775252      1.468225      1.740474
  H      4.416209     -0.580291     -1.756694
  H      4.254370     -0.798876      2.365257
  C     -0.576590     -1.737627     -1.314396
  C      0.856026     -1.786470     -1.395092
  C      1.311872     -1.984723     -0.105404
  C     -0.894393     -1.908413      0.018817
  O      0.249621     -2.066304      0.763829
  H     -1.289732     -1.574799     -2.119277
  H      1.479885     -1.677007     -2.279492
  H     -1.829195     -1.929629      0.572792
  H      2.298224     -2.084061      0.338696
  C     -0.800751      3.308574      0.135176
  C     -0.304374      2.251913      0.973698
  C      0.125944      1.250306      0.125592
  C     -0.630377      2.874735     -1.166419
  O     -0.061839      1.625044     -1.181111
```

```
 H     -1.229016      4.261646      0.439576
 H     -0.274870      2.221511      2.061198
 H     -0.839234      3.314005     -2.138207
 H      0.586043      0.279066      0.273383
---------------------------------------------------
```
